# The influence of microvascular injury on native T1 and T2* relaxation values after acute myocardial infarction: implications for non-contrast-enhanced infarct assessment

**DOI:** 10.1007/s00330-017-5010-x

**Published:** 2017-08-18

**Authors:** Lourens F. H. J. Robbers, Robin Nijveldt, Aernout M. Beek, Paul F. A. Teunissen, Maurits R. Hollander, P. Stefan Biesbroek, Henk Everaars, Peter M. van de Ven, Mark B. M. Hofman, Niels van Royen, Albert C. van Rossum

**Affiliations:** 1 Department of Cardiology, VU University Medical Centre, ZH 5F012, De Boelelaan 1117, 1081 HV Amsterdam, The Netherlands; 2 Department of Clinical Epidemiology and Biostatistics, VU University Medical Centre, Amsterdam, The Netherlands; 3 Department of Physics and Medical Technology, VU University Medical Centre, Amsterdam, The Netherlands

**Keywords:** Cardiovascular Magnetic Resonance imaging, Acute myocardial infarction, T1 mapping, T2* mapping, Microvascular injury

## Abstract

**Objectives:**

Native T1 mapping and late gadolinium enhancement (LGE) imaging offer detailed characterisation of the myocardium after acute myocardial infarction (AMI). We evaluated the effects of microvascular injury (MVI) and intramyocardial haemorrhage on local T1 and T2* values in patients with a reperfused AMI.

**Methods:**

Forty-three patients after reperfused AMI underwent cardiovascular magnetic resonance imaging (CMR) at 4 [3-5] days, including native MOLLI T1 and T2* mapping, STIR, cine imaging and LGE. T1 and T2* values were determined in LGE-defined regions of interest: the MI core incorporating MVI when present, the core-adjacent MI border zone (without any areas of MVI), and remote myocardium.

**Results:**

Average T1 in the MI core was higher than in the MI border zone and remote myocardium. However, in the 20 (47%) patients with MVI, MI core T1 was lower than in patients without MVI (MVI 1048±78ms, no MVI 1111±89ms, p=0.02). MI core T2* was significantly lower in patients with MVI than in those without (MVI 20 [18-23]ms, no MVI 31 [26-39]ms, *p*<0.001).

**Conclusion:**

The presence of MVI profoundly affects MOLLI-measured native T1 values. T2* mapping suggested that this may be the result of intramyocardial haemorrhage. These findings have important implications for the interpretation of native T1 values shortly after AMI.

***Key points*:**

• Microvascular injury after acute myocardial infarction affects local T1 and T2* *values*.

• *Infarct zone T1 values are lower if microvascular injury is present*.

• T2* *mapping suggests that low infarct T1 values are likely haemorrhage*.

• T1 and T2* *values are complimentary for correctly assessing post*-*infarct myocardium*.

**Electronic supplementary material:**

The online version of this article (doi:10.1007/s00330-017-5010-x) contains supplementary material, which is available to authorized users.

## Introduction

Cardiovascular Magnetic Resonance (CMR) imaging with native T1 mapping is a novel technique that allows direct voxelwise measurement of T1 values within the myocardium and has been proposed as a new tool for depicting the infarcted myocardium, without the need for contrast agents, thus providing an alternative for the use of gadolinium in patients with renal impairment [1, 2]. While some studies show a relation between increased myocardial T1 mapping values and adverse outcome, recent studies suggest that decreased values in the MI core are associated with worse outcome [3, 4]. Studies using late gadolinium enhancement (LGE) have shown that the infarcted myocardium consists of heterogeneous tissue with different gradations of injury, ranging from ruptured myocytes to more severely damaged myocardium, containing haemorrhage and/or microvascular obstruction, better described as microvascular injury (MVI) [5–9]. The influence of this infarct heterogeneity on local T1 values has not yet been fully elucidated. In ST-elevation myocardial infarction (STEMI), T1 values have been shown to increase due to the ischaemic damage and oedema formation [3], but the presence of MVI due to ischaemia-reperfusion induced myocardial haemorrhage may alter T1-related parameters, which may be detected with T2* mapping tools [4, 10]. These conflicting changes may well be a potential pitfall in the interpretation of T1 mapping parameters. The aim of this study was to assess whether local T1 and T2* values change within the myocardial tissue early after reperfused STEMI and whether these changes are altered if local haemorrhage is present. We hypothesised that the presence of microvascular injury (MVI) significantly affects local T1 and T2* values to warrant an adjustment in interpreting T1 and T2* values, if MVI is present.

## Methods

### Patient population

This single-centre study was approved by our local institutional review board and is in concordance with the Declaration of Helsinki. Written informed consent was obtained from all patients. Between December 2011 and February 2013, 60 consecutive patients presenting with a first STEMI were enrolled. This study is a substudy of our main trial, from which we recently published our main findings [11]. In short, patients were eligible for the study if they were admitted within 6 hours after the onset of symptoms with persisting ST-segment elevation on 12-lead electrocardiography (ECG) and if they underwent a successful primary PCI. Exclusion criteria were hemodynamic instability (Killip classes III and IV)[12, 13], prior myocardial infarction in the culprit coronary artery, prior coronary artery bypass graft (CABG) surgery or general contraindications for CMR. All patients were treated with medication according to current ESC guidelines [14].

Of the 60 participating patients, eight were excluded for this substudy. In one patient, the MRI examination was terminated due to software malfunction halfway during the acquisition, upon which the patient refused further MRI examination. Four patients refused informed consent for CMR because of anxiety or claustrophobia and two patients did not fit into the CMR scanner due to obesity. In one patient, a proximal dissection of a coronary artery occurred during angiography.

### Cardiovascular Magnetic Resonance

CMR was performed between 4-6 days after PCI using a clinical 1.5 Tesla MR-scanner (Magnetom Avanto, Siemens, Erlangen, Germany) with a dedicated 12-element phased-array cardiac receiver coil. A segmented T2 weighted turbo spin echo (T2w) sequence with fat suppression (Short-tau inversion recovery, STIR) was performed in a short-axis orientation with full LV coverage from mitral valve annulus to the apex. Using the STIR images, the centre of the infarcted myocardium containing oedema (defined as regional high signal) and, if present, haemorrhage (defined as attenuated signal within the area of high signal intensity[15–17]) was visually identified. At this specific slice position, a native T1 measurement was performed with a Modified Look-Locker Inversion-recovery (MOLLI) sequence (typical parameters: single breath-hold, voxel size 2.1x2.1x8 mm, field-of-view 360-400 mm, time of repetition 2.2 ms, echo time 1.1 ms, 11 inversion delays obtained in a 3-3-5 scheme in 17 heartbeats [2, 18]. Directly after the T1 measurement, a T2* measurement was acquired at the same slice position (typical parameters, voxel size 1.6x3.1x10 mm, field-of-view 400 mm, TR 32 ms, 12 echos, TE range of 2.6-30 ms) [19]. After administration of 0.2 mmol/kg Gd-DOTA (Dotarem, Guerbet, Villepinte, France), functional imaging was performed by using a retrospectively ECG-gated steady-state free precession (SSFP) cine imaging sequence with breath-holding at similar short-axis slice positions as the STIR images. At least 10 minutes after contrast administration, LGE images were acquired, using a 2-dimensional segmented inversion-recovery spoiled gradient-echo pulse sequence, with individual adjustment of the inversion time to suppress the signal of normal myocardium.

T1 measurements, T2* measurements, cine, STIR and LGE images within one examination were matched by slice position.

### CMR analysis and definitions

Analysis was performed with dedicated software (QMass MR v.7.5, Medis, Leiden, the Netherlands). Left ventricular volumes, ejection fraction (EF), end-diastolic myocardial mass and infarct size were calculated as previously described [20]. Infarct size was calculated from the LGE images by using the full-width at half-maximum (FWHM) technique [21]. MVI, defined as a hypo-intense region within the hyperenhanced infarcted myocardium, was incorporated in the infarcted area. Volumes, mass and infarct size were indexed for body surface area. Additionally, infarct size was also expressed as % of the LV myocardium by dividing it by end-diastolic mass.

**Fig. 1:**
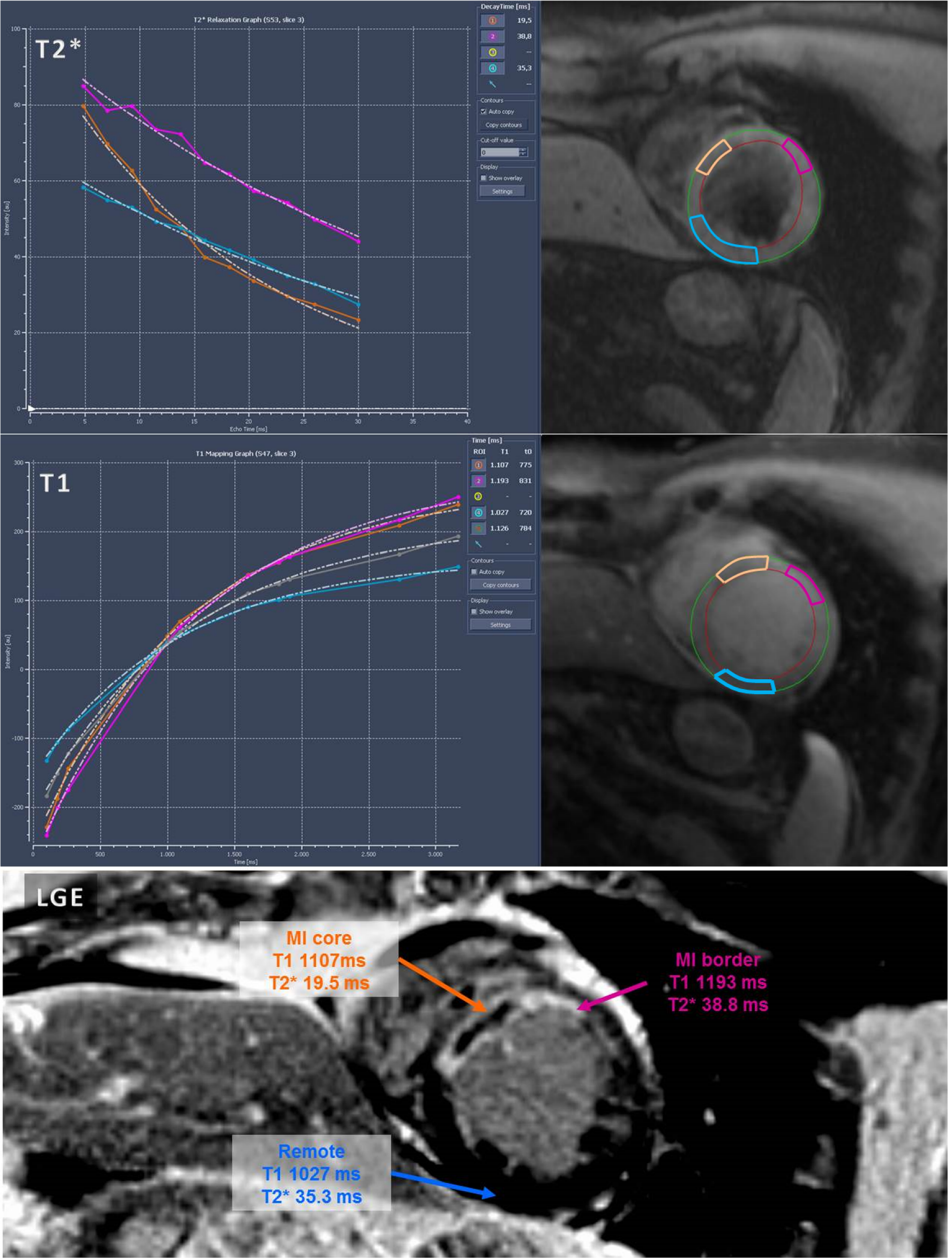
T2* maps, T1 maps and the corresponding LGE image, showing the different regions; MI core (orange), the adjacent MI border zone (pink) and remote myocardium (blue). MVI=Microvascular Injury, ms=millisecond

By matching the T1 and T2* images with the LGE images, three regions of interest (ROI) were visually identified in the left ventricular myocardium as follows: core: the infarcted area with the largest extent of transmural hyperenhancement, including MVI (when present); border zone: the adjacent infarcted area with hyperenhancement on LGE imaging, but without MVI; remote: a myocardial area in a different coronary territory without wall motion abnormalities, oedema or contrast enhancement. After matching the LGE images with the corresponding T1 and T2* maps by slice position, measurements were performed on the T1 and T2* maps in the LGE-defined regions of interest. On the T1 maps and T2* maps, ROI contours were drawn in the area of interest as defined on LGE images. Between the maps, contours were copied to keep the ROI positions in similar positions as much as possible, with manual correction for motion. From the ROIs, the T1 relaxation values were assessed from the MOLLI images as previously described [18]. The calculated values were corrected for heart rate dependency of the technique by the formula $T{1}_{corrected}=T{1}_{raw}-\left(2.7\times \left[ HR-70\right]\right)$ [22]. T2* values were determined using a single exponential fit of the signal intensities versus echo time. An example of the matching and the T1 and T2* analyses is shown in Fig. 1. All CMR analyses were performed by an experienced CMR reader (LR, 6 years of CMR experience), blinded to all patient data and outcomes.

### Statistical analysis

Categorical data are presented as frequencies (percentage) and continuous data as mean ± SD for normally distributed variables or median with [IQR]. Normally distributed variables were compared between groups using Student’s T-tests. Log-transformation was applied for the T2* relaxation values to achieve a normal distribution. Pearson correlation coefficients were calculated to quantify the strength of the association between variables. Intra- and inter-observer variability was tested in a sample of the studies by using an intraclass correlation coefficient with two-way measures and absolute agreement. For comparison of infarct core, MI border zone and remote T1 and T2* values, a repeated measures ANOVA was used with post-hoc Bonferroni correction for pairwise comparisons. Post-hoc tests were only performed in case of a significant overall effect between the three regions. Differences in maximal Creatin Kinase MB-fraction (CK-MB) levels were compared between groups using the non-parametric Mann-Whitney U tests as skewness could not be resolved by a transformation. All *p*-values are two-sided and statistical significance was set at *p*<0.05. Statistical analysis was done with the Statistical Package for Social Sciences software (IBM SPSS Statistics 20 for Windows).

## Results

### General characteristics of the population

Of the 52 patients included, nine were excluded from analysis because of insufficient quality of either T1 or T2* maps, which was mainly caused by the length of the breath hold. Exclusion was performed in consensus by two experienced readers. The maximal CK-MB levels of excluded patients were not significantly different from the levels of included patients.

All 43 patients underwent CMR examination at a median of 4 [3-5] days after PCI. Patient demographics are listed in Table 1 and all functional and mapping parameters are displayed in Tables 2 and 3. Twenty patients (47%) had MVI on the LGE images. Patients with MVI had significantly lower LVEF (MVI: 46±6% vs. no MVI: 55±8%, *p*<0.001) and larger infarct areas (MVI: 25±11% vs. no MVI: 10±7%, *p*<0.001).

**Table 1:** General demographics of patients

	Total (*n*=43)	
Parameter		
General demographics		
Age (years)	59 ±9	
Body Mass Index (g/m^2^)	27 ±3	
Infarct-related artery		
LAD	26	(61%)
RCA	12	(28%)
LCx	5	(12%)
Male gender (%)	33	(77%)
Type 2 diabetes (%)	4	(9%)
Hypertension (%)	7	(16%)
Smoking (%)	35	(81%)
Hypercholesterolemia (%)	7	(16%)
Positive family history for CAD (%)	22	(51%)
Medication at discharge		
Aspirin (%)	42	(98%)
Thienopyridine (%)	43	(100%)
Coumadin (%)	4	(10%)
Beta blocker (%)	39	(95%)
Statin (%)	43	(100%)
Ace inhibitor (%)	31	(74%)
Angiotensin receptor blocker (%)	4	(10%)
Aldosterone receptor blocker (%)	4	(10%)
Calcium antagonist (%)	1	(2%)
Diuretics (%)	4	(10%)

LAD=Left Anterior Descending coronary artery, RCA=Right Coronary Artery, LCx=Left Circumflex coronary artery

**Table 2:** Functional characteristics of patients with and without MVI

	Total (*n*=43)	MVI (*n*=20)	No MVI (*n*=23)	*p*-value
Functional parameters				
Indexed end-diastolic volume (ml/m^2^)	91 ± 19	95 ± 14	88 ± 23	0.23
Indexed end-systolic volume (ml/m^2^)	46 ± 17	52 ± 12	41 ± 20	0.04
LV Ejection fraction (%)	51 ± 8	46 ± 6	55 ± 8	<0.001
Infarct size (% of LV)	17 ± 12	25 ± 11	10 ± 7	<0.001

LV=Left Ventricle, Functional parameters are compared between patients with MVI and without MVI using Student’s T-tests

**Table 3:** Mapping characteristics of patients with and without MVI

	Total (*n*=43)	MVI (*n*=20)	No MVI (*n*=23)	*p*-value
Mapping parameters				
Heart rate during acquisition (bpm)	65 ± 12	70 ± 13	61 ± 10	0.01
T1 relaxation				
MI Core (ms)	1081 ± 89	1048 ± 78	1111 ± 89	0.02
MI Border (ms)	1093 ± 85	1129 ± 74	1063 ± 83	0.009
Remote (ms)	977 ± 61	991 ± 38	964 ± 75	0.16
T2* relaxation				
MI Core (ms)	25 [20-34]	20 [18-23]	31 [26-39]	<0.001
MI Border zone (ms)	30 [26-36]	30 [26-36]	30 [26-36]	0.74
Remote (ms)	27 [23-32]	28 [24-34]	27 [21-30]	0.23

MVI=Microvascular Injury. T1 and T2* values are compared between patients with MVI and without MVI using Student’s T-tests after log-transformation of T2* values

### T1 and T2* mapping values in all patients

Intra-observer variability (T1 values: ICC = 0.62, T2* values: ICC = 0.73, *p*=0.002) and inter-observer variability (T1 values: ICC = 0.92, T2* values: ICC = 0.69) were good (Appendix). For the group as a whole, mean T1 was found to differ between the MI core and remote zone (MI core: 1081±89 ms; MI border zone 1093±85 ms; remote 977±61 ms), with post-hoc test revealing averaged MI core and MI border zone T1 to be significantly longer than T1 in the unaffected, remote myocardium (both *p*-values <0.001). T1 did not differ between MI core and MI border zone (*p*=0.50). T2* MI core values were also found to differ between the zones (MI core: 25 [20-34] ms; MI border: 30 [26-36] ms; remote: 27 [23-32] ms; overall *p*=0.003), with post-hoc test showing T2* to be lower in the MI core zone and remote zone compared to the MI border zone (*p*= 0.001 and *p* = 0.006, respectively). T2* values did not differ significantly between the MI core and remote zone (*p*=0.32).

### T1 and T2* mapping values in patients with and without MVI

**Fig. 2:**
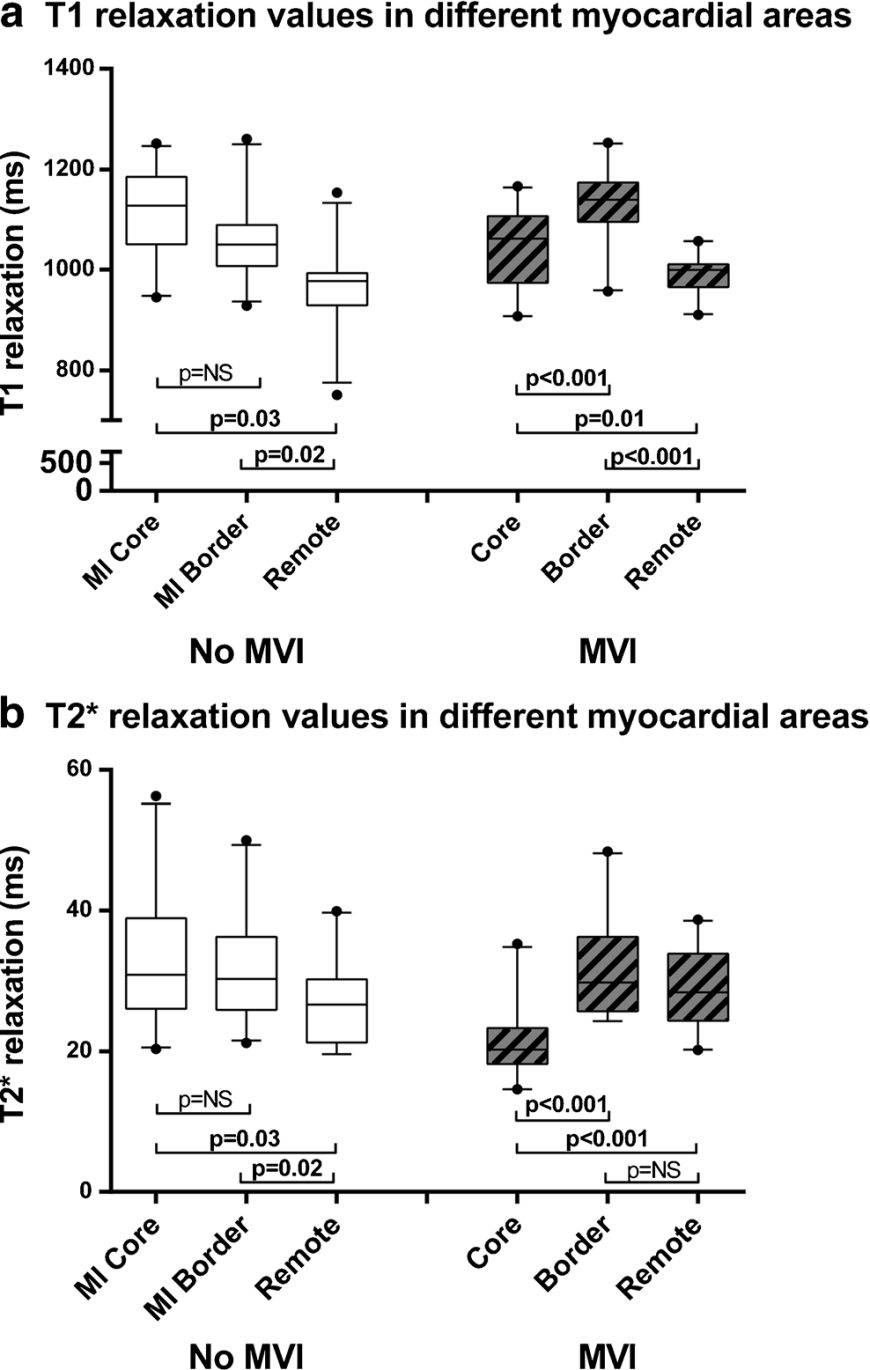
**A and B** T1 (A) and T2* (B) values for the myocardial areas of interest between patients with MVI and patients without MVI. MVI=Microvascular Injury, ms=millisecond. Whiskers represent 5th-95th percentile. *p*-values for T2* values were calculated after log-transformation. Comparisons between different areas was done with repeated measures ANOVA with post-hoc Bonferroni correction. Test of between-subject effects for T1: all *p*-values <0.001, for T2: *p*=0.03 without MVI, *p*<0.001 with MVI

In patients with MVI, the MI core T1 was significantly shorter than in patients without MVI (MVI: 1048±78 ms, vs. no MVI: 1111±89 ms, *p*=0.02). Patients with MVI also had lower MI core T2* values (MVI: 20 [18-23] ms vs. no MVI: 31 [26-39] ms, *p*<0.001). MI border zone T1 was significantly longer in patients with MVI than in patients without MVI (MVI: 1129±74 ms, vs. no MVI: 1063±83 ms, *p*=0.009), but T2* values did not differ (MVI: 30 [26-36] ms vs. no MVI: 30 [26-36] ms, *p*=0.74). Figure 2A and B show the differences in T1 (2A) and T2* (2B) values between the regions for patients with and without MVI.

## Discussion

After reperfusion of acutely ischaemic myocardium, previous studies showed that average T1 and T2* values change in the affected area as the result of infarction-related oedema [1, 4, 8, 23].Our study confirmed these findings and shows that patients with microvascular injury have decreased T1 and T2* values in the MI core. This has implications for the interpretation of native T1 mapping values shortly after AMI as, without the proper use of T2* mapping, myocardium with MVI may be incorrectly classified as normal, unaffected myocardium.

LGE studies have shown that microvascular injury may affect up to 30 to 50% of patients with AMI [24–26]. MVI is associated with increased infarct size, and is a well-established predictor of impaired functional recovery, remodelling and increased incidence of major adverse cardiac events [25–27]. Histological studies of MVI show that intramyocardial haemorrhage is a major component of the injury, due to disruption of the microvasculature and extravasation of blood cells upon reperfusion [7, 28–30]. A number of erythrocyte haemoglobin breakdown products, most notably deoxyhaemoglobin and methaemoglobin, induce paramagnetic effects, effectively altering the tissue relaxation times in the area of MVI [31, 32]. From brain ischaemia studies, it is known that local T1 and T2 relaxation time values shorten due to fibrin clot formation and retraction, erythrocyte dehydration, and changes in the water hydration layer due to resorption of water from the protein solutions in the haemorrhagic area [31, 33]. Previous studies already suggested that T1 values might be suggestive of the severity of injury in the reperfused MI core [3, 4]. Dall’armellina et al. showed how T1 values rise in more severe forms of myocardial infarction, but did not incorporate the presence or absence of MVI [3]. Carrick et al. demonstrated in a large cohort that decrease in T1 values in the MI core is associated with worse outcome and postulated that it is most likely caused by haemorrhage [4]. However, no cine images or STIR-targeted T1 maps of the MI core were made. Our study confirms the aforementioned findings and demonstrates that T2* mapping corroborates that the changes in T1 values are likely caused by the effects of haemorrhage [7]. T2* is considered even more sensitive to the effects of the haemoglobin breakdown and haemorrhage than T2-weighted imaging [34]. During spin echo signal creation in T2 weighted imaging, magnetic spins are refocused and rephased using 180 degree RF pulses prior to signal detection, thus correcting the loss of signal due to static susceptibility influences. T2* imaging on the other hand, is sensitive to static susceptibility effects and strongly decreases due to iron in haemoglobin breakdown products. T2* mapping is a well-established technique to detect myocardial iron deposition in transfusion dependent patients, [35–37] with values below 20 ms considered to be abnormal. It has been shown that lower T2* values are associated with lower LVEF and adverse remodelling [8]. We confirmed that T2* values in the MI core of patients with MVI are around this lower limit of normal, and clearly lower than in patients without MVI, or in remote areas. Comparable to T2 relaxation, T2* increases in the presence of myocardial oedema, which explains the higher values in the MI border zones of the infarction.

The magnetic susceptibility effects related to the presence of haemorrhage in the MI core are a potential pitfall in the interpretation of T1 measurements in patients with a recently reperfused AMI. MI border zone T1 was higher in patients with MVI, reflecting more severe myocardial injury and inflammation. Interestingly, previous studies have found that increased T1 values were associated with more severe myocardial injury and less functional improvement [3, 38]. In these studies, the T1 relaxation times were averaged for the entire myocardium, and the high T1 found in the MI border zone of the infarct may have more than offset the lower values in the MI cores of the patients with MVI. Our results support earlier findings that local differences in T1 relaxation due to the presence of MVI and haemorrhage need to be considered [4]. A wide range of T1 values was found for remote myocardium, which may be explained by differences in myocardial perfusion [39]. However, further studies are needed to investigate this phenomenon and its cause. An inherent limitation of MOLLI acquisitions are its relatively long breath-holds (17 heart beats) which make it sensitive to motion artefacts. This led to an exclusion of nine of our 52 participating patients, making it the most common reason for study exclusion. This could, theoretically, be tackled by using a shorter acquisition technique, such as ShMolli [40, 41]. However, as remote T1 values were similar in both patient groups, a significant measurement error seems unlikely. Although our study group was relatively small, T2* values showed a correlation with volumes and ejection fraction where T1 values did not. This supports earlier findings that T2* values may confer additional prognostic value [8]. Further studies in larger study populations are necessary to define the prognostic significance of T1 and T2* values.

### Limitations

As our study group was relatively small, subgroup analyses on differences in T1 and T2* values for infarcts in different coronary territories was not possible. Earlier findings suggest that T2* values may confer prognostic value on cardiac function [8]. However, the numbers in our study are too small for creating a reliable model with proper correction for confounding factors. Further studies with a larger number of patients are necessary to assess the prognostic significance of T1 and T2* values.

MOLLI T1 mapping causes a systematic underestimation of T1 relaxation in tissues with short T2 values [42]. As the T2 and T2* relaxation are interdependent, it is expected that MOLLI underestimates T1 relaxation in tissues with short T2* values. Whether the shortened T1 values in the areas with MVI are based on a MOLLI-specific artefact or ‘genuinely’ shortened T1 values remains to be investigated using a technique like SACHA, [40, 43] but for clinical purposes the presence of MVI should be considered when using MOLLI-measured T1 maps, as the MOLLI technique is one of the most widely applied techniques for myocardial T1 assessment today [44]. Additionally, it should be noted that the MOLLI sequence parameters have been improved in the last years [40, 41]. However, our study was performed with the MOLLI sequence parameters that were used at the start of the study, in 2011 [40].

Finally, we did not assess myocardial T2 relaxation in our study, which poses a limitation to our data as T2 mapping would have given us additional insight in the tissue characteristics. However, a robust version of this sequence was not available at the time of initiation.

## Conclusion

In conclusion, infarcted areas of MVI within the reperfused myocardium have shorter T1 relaxation and T2* relaxation than infarcted areas without MVI. In the adjacent MI border zone; T1 relaxation is longer in patients with MVI. This can be a potential pitfall in the interpretation or quantification of native T1 mapping values using the MOLLI technique, without the use of T2* mapping. Combining T1 and T2* mapping may provide a new approach to differentiate between normal myocardium, infarcted myocardium, and infarcted myocardium with microvascular injury, without the use of contrast agents, but further studies are warranted to assess the diagnostic value of this potential new biomarker.

### Electronic supplementary material

Below is the link to the electronic supplementary material.

ESM 1 (XLS 30 kb)

## Figures and Tables

**Fig. 3:**
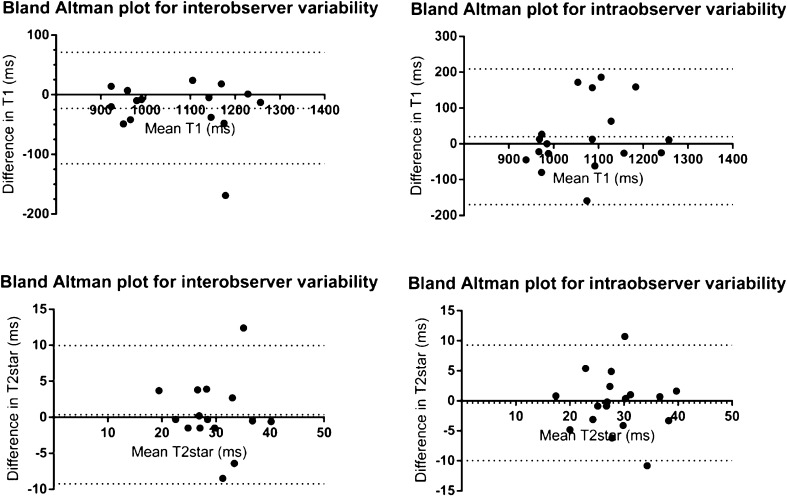
Bland Altman plots for inter- and intra-observer variability of T1 and T2* measurements
